# Crystal structure, DFT calculation, Hirshfeld surface analysis and energy framework study of 6-bromo-2-(4-bromo­phen­yl)imidazo[1,2-*a*]pyridine

**DOI:** 10.1107/S2056989019013410

**Published:** 2019-10-03

**Authors:** Hussien Ahmed Khamees, Kumara Chaluvaiah, Nasseem Ahmed El-khatatneh, Ananda Swamynayaka, Kwong Huey Chong, Jagadeesh Prasad Dasappa, Mahendra Madegowda

**Affiliations:** a Department of Studies in Physics, Manasagangotri, University of Mysore, Mysuru 570 006, Karnataka, India; b Department of Chemistry, Mangalore University, Mangalagangothri, Mangaluru 574 199, Karnataka, India; c Department of Chemistry, Faculty of Science, Universiti Putra Malaysia 43400, UPM Serdang, Selangor Darul Ehsan, Malaysia

**Keywords:** crystal structure, imidazole-pyridine derivative, π–π inter­actions, DFT calculation, Hirshfeld surface analysis, energy framework, frontier mol­ecular orbitals

## Abstract

The title imidazo[1,2-*a*] pyridine derivative, C_13_H_8_Br_2_N_2_, was synthesized *via* a single-step reaction method. The title mol­ecule is planar, showing a dihedral angle of 0.62 (17)° between the phenyl and the imidazo[1,2-*a*] pyridine rings. An intra­molecular C—H⋯N hydrogen bond with an *S*(5) ring motif is present. In the crystal, a short H⋯H contact links adjacent mol­ecules into inversion-related dimers. The dimers are linked in turn by weak C—H⋯π and slipped π–π stacking inter­actions, forming layers parallel to (110). The layers are connected into a three-dimensional network by short Br⋯H contacts. Two-dimensional fingerprint plots and three-dimensional Hirshfeld surface analysis of the inter­molecular contacts reveal that the most important contributions for the crystal packing are from H⋯Br/Br⋯H (26.1%), H⋯H (21.7%), H⋯C/C⋯H (21.3%) and C⋯C (6.5%) inter­actions. Energy framework calculations suggest that the contacts formed between mol­ecules are largely dispersive in nature. Analysis of HOMO–LUMO energies from a DFT calculation reveals the pure π character of the aromatic rings with the highest electron density on the phenyl ring, and σ character of the electron density on the Br atoms. The HOMO–LUMO gap was found to be 4.343 eV.

## Abstract 2

The mol­ecular system displays a planar conformation between the phenyl and imidazo[1,2-*a*] pyridine rings. Weak C—H⋯π and π–π inter­actions as well as short contacts consolidate the three-dimensional network structure.

## Chemical context   

Five-membered heterocyclic compounds comprising a nitro­gen atom and at least one other non-carbon atom (*i.e.* nitro­gen, sulfur, or oxygen) as part of the ring are known as azoles. To date, numerous azoles have found a wide range of applications in various fields, including agriculture (Berger *et al.*, 2017 ▸), and because of their biological activities (Pozharskii *et al.*, 2011 ▸; Kumbar *et al.*, 2018 ▸). Among the various classes of azoles, the imidazole moiety with two nitro­gen atoms is extremely common in nature and forms the core of many biomolecules (Chopra & Sahu, 2019 ▸) and synthetic drugs (Pozharskii *et al.*, 2011 ▸). Furthermore, pyridine and its derivatives are present in many important compounds, including pharmaceuticals, vitamins (Al-Ghorbani *et al.*, 2016 ▸) and drugs, acting as anti­microbial, anti­viral, anti­oxidants, anti­diabetic, anti-malarial, anti-inflammatory or anti­amoebic agents, as well as psychopharmacological antagonists (Altaf *et al.*, 2015 ▸). Hence, the combination of pyridine and imidazole derivatives has been proven to result in highly active agents in diverse biological fields that include anti­cancer (Kamal *et al.*, 2014 ▸; Mantu *et al.*, 2016 ▸), anti-HIV (Bode *et al.*, 2011 ▸), anti­bacterial (Rival *et al.*, 1992 ▸) and anti-inflammatory (Rupert *et al.*, 2003 ▸) properties. In addition, such a combination showed significant activity against the human *cytomegalo* virus and the *varicella-zoster* virus (Gueiffier *et al.*, 1998 ▸; Mavel *et al.*, 2002 ▸).
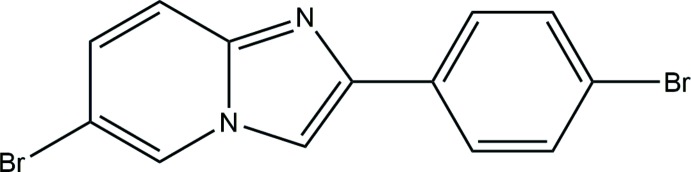


In this context, we synthesized a new imidazo[1,2-*a*] pyridine derivative, C_13_H_8_Br_2_N_2_, and report herein its mol­ecular and crystal structure, as well as the qu­anti­fication of supra­molecular inter­actions by Hirshfeld surface analysis. This study is supplemented by DFT calculations and a comparison of structural details with related compounds.

## Structural commentary   

**Figure 1:**
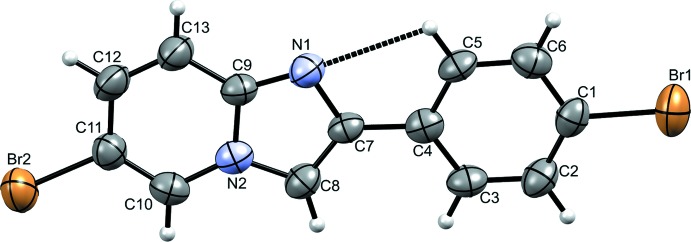
The mol­ecular structure of the title compound with displacement ellipsoids drawn at the 50% probability level. The intra­molecular C—H⋯N hydrogen bond forming an *S*(5) ring motif is shown with dashed lines.

The mol­ecular structure of the title compound is depicted in Fig. 1 ▸. The mol­ecular system is planar, showing a dihedral angle of 0.62 (17)° between the phenyl ring (C1–C6) and the imidazo[1,2-*a*] pyridine ring system (C7–C13,N1,N2). The torsion angles about the terminal bromine atoms, Br1 and Br2, are 177.3 (3)° (Br1—C1—C6—C5) and −178.9 (4)° (Br2—C11—C12—C13), respectively. The planar arrangement between the two rings enables an intra­molecular C—H⋯N inter­action (Fig. 1 ▸, Table 1 ▸) forming an *S*(5) ring motif (Tan & Tiekink, 2019 ▸). The Br1—C1 and Br2—C11 bond lengths are 1.886 (4) Å and 1.880 (4) Å, respectively, in good agreement with structures comprising bromo­phenyl moieties (Zhang & Hu, 2005 ▸; Arif Tawfeeq *et al.*, 2019 ▸). The N1=C9 bond is slightly longer than similar bonds of reported imidazo[1,2-*a*] pyridine structures (see §7 for a listing of these structures), which may be attributed to the presence of the intra­molecular bond (H5⋯N1). Overall, the bond lengths and angles of the phenyl ring and the imidazo[1,2-*a*]pyridine ring system are in normal ranges and compare well with those of other imidazo[1,2-*a*]pyridine derivatives (Zhang *et al.*, 2005 ▸; Dhanalakshmi *et al.*, 2018 ▸).

## Supra­molecular features   

**Figure 2:**
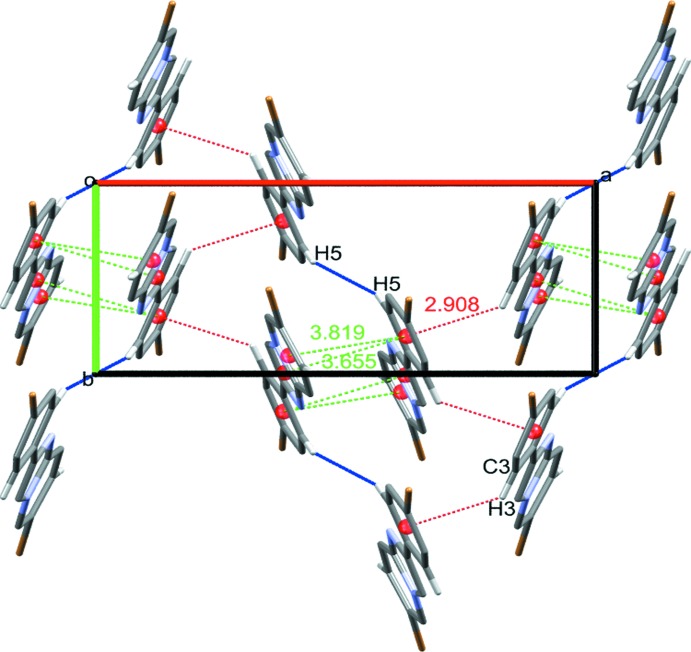
The crystal packing of the title compound in a view along [001], showing inter­actions in the sheets. H5⋯H5 short contacts are represented as blue dashed lines, C3—H3⋯*Cg*3 inter­actions as red dashed lines (slippage 1.676 Å) and *Cg*3⋯*Cg*1 and *Cg*3⋯*Cg*2 inter­actions as light-green dashed lines.

**Figure 3:**
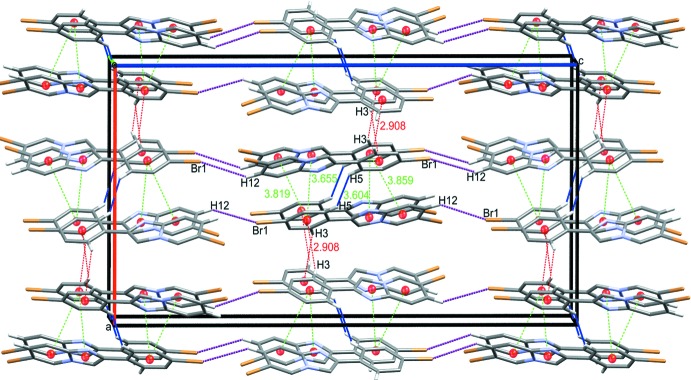
The three-dimensional supra­molecular network of the title compound viewed approximately along [010].

The crystal packing is mainly based on short contacts and weak π–π inter­actions, similar to reported structures with the same kind of terminal bromine atoms (Arif Tawfeeq *et al.*, 2019 ▸). In the title compound, two inversion-related mol­ecules are linked by a short H5⋯H5(1 − *x*, 2 − *y*, *z*) contact (Fig. 2 ▸). These dimers are connected to each other through C—H⋯π inter­actions (Table 1 ▸), forming sheets propagating parallel to (110). Slipped π–π stacking inter­actions [*Cg*3⋯*Cg*1(−*x*, 1 − *y*, −*z*) = 3.655 (2) Å, slippage of 0.885 Å; *Cg*3⋯*Cg*2(−*x*, 1 − *y*, −*z*) = 3.819 (2) Å, slippage of 1.473 Å], where *Cg*1*, *Cg**2 and *Cg*3 are the centroids of the imidazole, pyridine and phenyl rings, respectively, are also present within these sheets (Fig. 2 ▸). Adjacent sheets are linked along [001] into a three-dimensional network through short contacts of 3.01 Å between Br1 and H12(*x*, 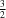 − *y*, 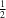 + *z*), forming *S*(11) chain motifs (Fig. 3 ▸).

## DFT study and FMOs   

Density functional theory (DFT) calculations were carried out by using the B3LYP basis set (Becke, 1993 ▸) at the highest basis set level of 6-311 ++G(d,p) in the *GAUSSIAN09* program (Frisch *et al.*, 2009 ▸). The DFT-optimized structure of the title compound is generally found to be in good agreement with the experimental data for all bond lengths and angles.

**Figure 4:**
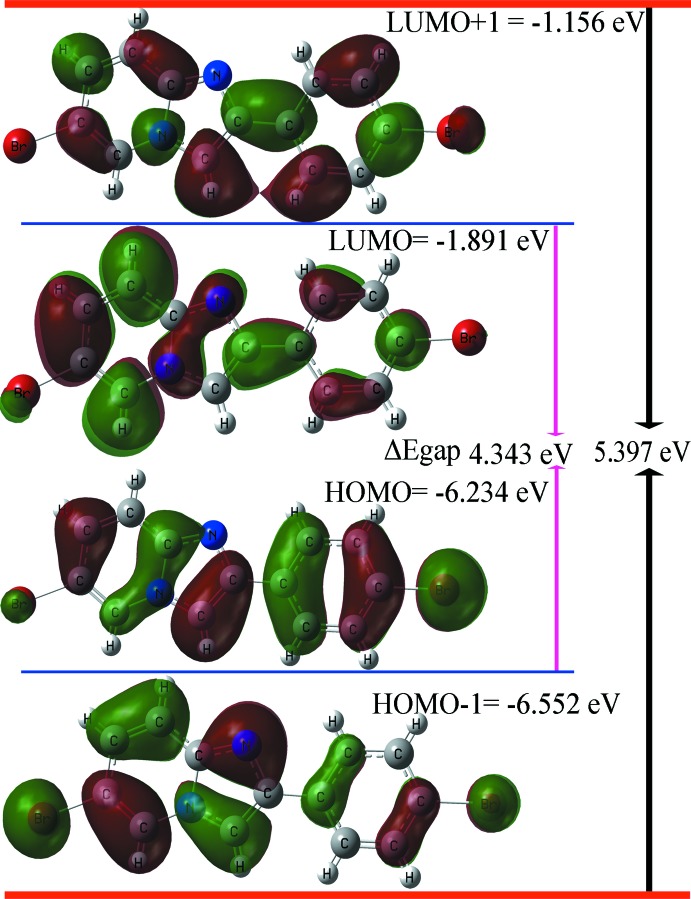
Electron distribution and mol­ecular orbital energies of HOMO-1, HOMO, LUMO and LUMO+1 of the title compound.

Frontier mol­ecular orbitals (FMOs) are useful to specify the distribution of electronic densities and other quantum chemical parameters including hardness (η), softness (ζ), chemical potential (μ), electrophilicity (ψ) and electronegativity (χ) by foreseeing the highest occupied mol­ecular orbitals (HOMO) and the lowest-unoccupied mol­ecular orbitals (LUMO), as well as the energy gap (*E*_g_ = *E*_H_ - *E*_L_) (Khamees *et al.*, 2018 ▸). The results of these calculations are compiled in Table 2 ▸, and orbital energy plots of (*E*_H_, *E*_H-1_) and (*E*_L_, *E*_L+1_) are depicted in Fig. 4 ▸. The HOMO (ground state) manifests the highest π characterization for phenyl ring (C1–C6) that displays bifurcated π–π stacking inter­actions as well as C—H⋯π inter­actions in the supra­molecular network, as discussed in Section 3. Pronounced σ character of the electron density is located on the two Br atoms, with the higher amount located on Br1. The other FMOs orbitals, *i.e*. HOMO-1, LUMO and LUMO+1, exhibit a mix of π and σ character on the rings with variations of the electron density distribution (Fig. 4 ▸). The HOMO–LUMO gap is 4.343 eV for the title compound.

## Hirshfeld surface analysis   

**Figure 5:**
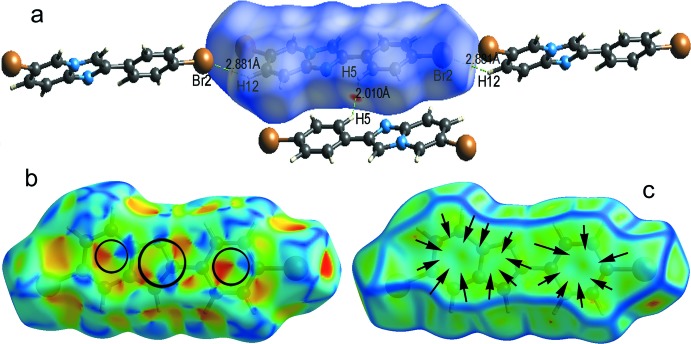
(*a*) Hirshfeld surface mapped over *d*_norm_ showing short contacts as green dashed lines, (*b*) shape-index map and (*c*) curvedness map showing regions of π–π inter­actions.

**Figure 6:**
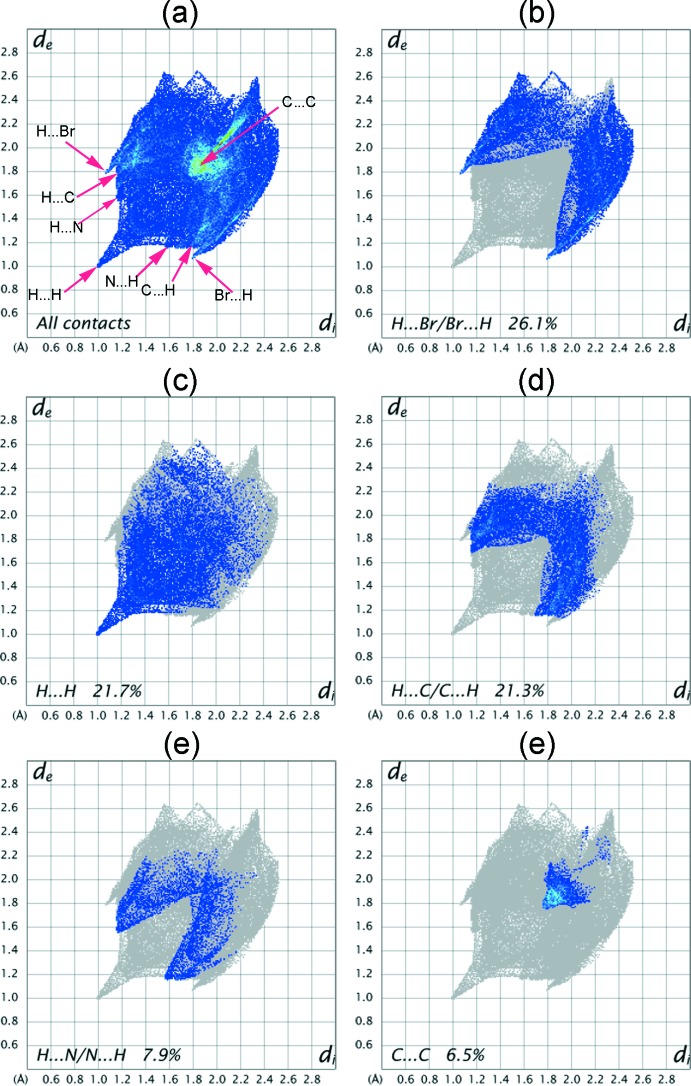
Two-dimensional fingerprint plots of the title mol­ecule with their relative contributions to the Hirshfeld surface.

The nature of inter­molecular inter­actions in the title compound has been computed by *CrystalExplorer17.5* (Turner *et al.*, 2017 ▸), using Hirshfeld surface analysis (Spackman & Jayatilaka, 2009 ▸) and two-dimensional fingerprint plots (McKinnon *et al.*, 2007 ▸). The *d*_norm_ plot was estimated *via* calculations of the external (*d*_e_) and inter­nal (*d*_i_) distances to the nearest nucleus and built over the volume of 363.34 Å^3^ and an area of 339.81 Å^2^, with scaled colour of −0.1544 (red) a.u. to 1.0479 (blue) a.u. (Fig. 5 ▸ *a*). The plots of shape-index and curvedness were generated in the range of −4.0 to 4.0 a.u. and −1.00 to 1.00 a.u., respectively, (Fig. 5 ▸ *b,c*). The medium dark and side pale-red spots on the Hirshfeld surface (Fig. 5 ▸ *a*) symbolize the H5⋯H5 and Br1⋯H12 short contacts, respectively. The two-dimensional fingerprint plot for all contacts is depicted in Fig. 6 ▸ *a*. The H⋯Br/Br⋯H contacts make the largest contribution (26.1%) to the Hirshfeld surface (Fig. 6 ▸ *b*). These contacts also make a significant contribution to the crystal packing as the distance between the atoms involved is slightly less than their van der Waals radii (*d*_i_ + *d*_e_ ≃ 3.01 Å). The inter­atomic contacts of H⋯H inter­actions generated 22.7% of the Hirshfeld surface (Fig. 6 ▸ *c*), showing a short spike at diagonal axes *d*_i_ + *d*_e_ ≃ 2.24 Å < 2.4 Å, denoting H⋯H short contacts with another significant effect on the mol­ecular packing. The two symmetrical broad wings in Fig. 6 ▸ *d* belong to H⋯C/C⋯H contacts that represent 21.3% of total surface and indicate the presence of C—H⋯π inter­actions in the crystal packing, where *d*_i_ + *d*_e_ ≃ 2.77 Å < 2.90 Å. The proportion of H⋯N/N⋯H contacts is 7.9% of the Hirshfeld surface (Fig. 6 ▸ *e*) and they appear as two close wings pointing at a distance greater than the van der Waals radii of N and H atoms (*d*_i_ + *d*_e_ > 2.75Å), with no significant contribution towards the crystal packing of the title mol­ecule. The small contribution of the C⋯C contacts (6.5%) to the Hirshfeld surface appears as an intense triangle (Fig. 6 ▸ *f*) at *d*_i_ + *d*_e_ ≃ 3.6 Å, indicating π–π stacking inter­actions in the crystal packing. This type of stacking inter­action appears as a flat region on the curvedness (Fig. 5 ▸ *c*) and also on the shape-index as red and blue triangles on the rings (Fig. 5 ▸ *b*), in particular on the phenyl ring (C1–C6). The contributions from other contacts have negligible effects on the packing.

## Energy framework   

**Figure 7:**
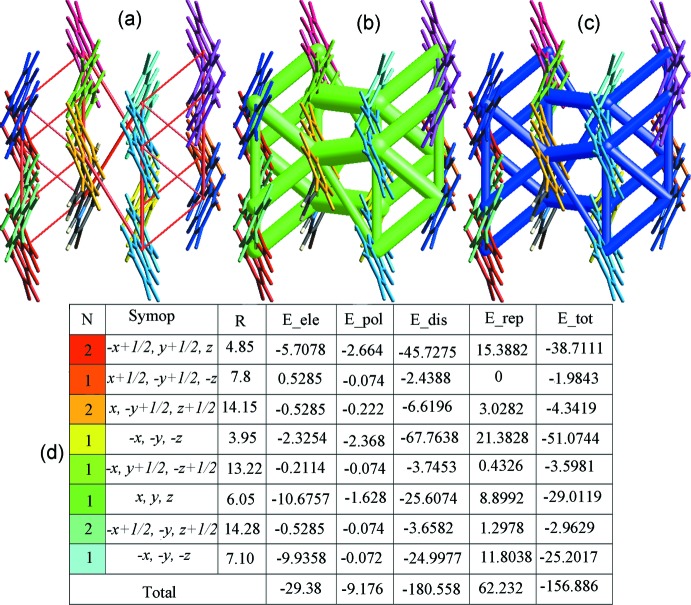
Energy framework of the title mol­ecules viewed along [001], showing: (*a*) electrostatic, (*b*) dispersion, (*c*) total energy force diagrams and (*d*) the details of inter­action with colour-coded, symmetry operation (Symop) and distances between mol­ecular centroids (*R*) in Å.

Qu­anti­fication of energy framework energies is considered a powerful method for understanding the topology of the overall inter­actions of mol­ecules in the crystal. This method allowed us to calculate and compare different energy components, *i.e.* repulsion (*E*_rep), electric (*E*_ele), dispersion (*E*_dis), polarization (*E*_pol) and total (*E*_tot) energy based on the anisotropy of the topology of pairwise inter­molecular inter­action energies. *CrystalExplorer17.5* (Turner *et al.*, 2017 ▸) was used to calculate the energy framework of the title compound by generating new wave functions using the DFT method under 3-21G basis set with exchange and potential functions (B3LYP) for a mol­ecular cluster environment for a 1×1×1 unit cell. The thickness of the cylinder radius indicates the grade of inter­actions and is directly related to the energy magnitude and offers information about the stabilization of the crystal packing. In order to avoid the crowdedness of less significant inter­action energies, we set the cylindrical radii with a cut-off value of 5 kJ mol^−1^ and a scale factor of 50 to all energy components. The benchmarked energies were scaled according to Mackenzie *et al.* (2017 ▸) while *E*_rep, *E*_ele, *E*_dis and *E*_pol were scaled as 0.618, 1.057, 0.740, 0.871, respectively (Edwards *et al.*, 2017 ▸). The results of the calculations revealed that dispersion inter­actions exhibit approximately chair-shaped energy topologies through the rings, having a maximum energy value of −180.558 kJ mol^−1^ (Fig. 7 ▸). The other energy components have values of 62.232 kJ mol^−1^, −29.38 kJ mol^−1^ and −9.176 kJ mol^−1^ for repulsion, electrostatic and polarization energies, respectively. The small value of electrostatic energy is attributed to the absence of classical hydrogen bonds. The total inter­action energy that resulted from all four main components is −156.886 kJ mol^−1^ (Fig. 7 ▸ *d*).

## Database survey   

36 structures containing the 2-phenyl­imidazo[1,2-*a*]pyridine moiety with different substituents were found in a search of the Cambridge Structural Database (CSD, version 5.40, last update May 2019; Groom *et al.*, 2016 ▸). The different substit­uents *R*_1_ (on the imidazo[1,2-*a*]pyridinyl ring) and *R*_2_ (on the phenyl ring) together with the dihedral angles between the mean planes of the corresponding imidazo[1,2-*a*]pyridinyl and phenyl rings (dihedral angle 1) are compiled in Table 3 ▸. By comparing the substitution positions, the structures can be divided into ‘3-(substituted)imidazo[1,2-*a*]pyridin­yl’ compounds and ‘non-3-(substituted)imidazo[1,2-*a*]pyridin­yl’ compounds. In general, the 3-(substituted)imidazo[1,2-*a*]pyridinyl compounds have a greater dihedral angle 1 values (12.0–47.5°). This may arise from steric repulsion between the 3-(substituted) group and the phenyl ring. However, there are four outliers (KABMIM, MIXZOJ, MONREO and ZUSSAJ) whose dihedral angle 1 values are lower than 10°. Most of the non-3-(substituted)imidazo[1,2-*a*]pyridinyl compounds have dihedral angle 1 values between 0.7 and 12.5°, which indicates that the imidazo[1,2-*a*]pyridinyl rings are close to coplanar to their attached phenyl rings. Here, the outlier is JEBZEY where the imidazo[1,2-*a*]pyridinyl ring is attached to a di-*ortho*-substituted isophthalo­nitrile ring. The dihedral angle 1 is 46.4° in this structure.

## Synthesis and crystallization   

5-Bromo­pyridin-2-amine (1.211 g, 0.007 mol) and phenacyl bromide (0.007 mol) were refluxed for 14 h in 50 ml of absolute ethanol. The progress of the reaction was monitored by thin layer chromatography using Merck alumina backed silica gel 60 F254. After completion of the reaction, the resulting product was poured into crushed ice to obtain a fine grained solid product that was filtered off, separated and dried. The crude product was then recrystallized from hot ethanol with a yield of ∼70%. The melting point of 345 K was determined in an open capillary and is uncorrected. IR (KBr, cm^−1^): 3080 (Ar C—H stretch), 2918 (aliphatic C—H stretch, 4-bromo­phenyl moiety), 1587 (C=N stretch), 1332 (C—N), 792 and 595 (C—Br). ^1^H NMR (400 MHz, DMSO, δ ppm): 7.37 (*d*, 1H, 5-bromo­pyridine moiety), 7.55 (*d*, 2H, 4-bromo­phenyl moiety), 7.56 (*d*, 1H, 5-bromo­pyridine moiety), 7.78 (*d*, 2H, 4-bromo­phenyl moiety), 8.37 (*s*, 1H, imidazole ring), 8.87 (*s*, 1H, 5-bromo­pyridine moiety).^13^C NMR (400 MHz, δ ppm): 145.13 (imidazo­pyridine carbon atom), 110.24, 119.82, 125.12 and 132.14 (four carbon atoms of 5-bromo­pyridine moiety), 123.12, 128.30, 132.11, and 132.32 (six carbon atoms of 4-bromo­phenyl moiety), 113.13 and 130.10 (two carbon atoms of imidazole ring). LC–Mass *m*/*z* 350 [M+], 352 [*M*+2], 354 [*M*+4]. Analysis calculated for C_13_H_8_Br_2_N_2_ (350): C, 44.36; H, 2.29; N, 7.96. Found: C, 44.31; H, 2.23; N, 7.92%.

## Refinement   

Crystal data, data collection and structure refinement details are summarized in Table 4 ▸. Hydrogen atoms were placed in calculated positions (C—H = 0.93 Å) and were included in the refinement in the riding-model approximation, with *U*_iso_(H) set to 1.2*U*_eq_(C). The reflection (002) was affected by the beam-stop and was removed from the refinement

## Supplementary Material

Crystal structure: contains datablock(s) I. DOI: 10.1107/S2056989019013410/wm5525sup1.cif

Structure factors: contains datablock(s) I. DOI: 10.1107/S2056989019013410/wm5525Isup2.hkl

Supporting information file. DOI: 10.1107/S2056989019013410/wm5525Isup3.cml

CCDC references: 1914069, 1914069

Additional supporting information:  crystallographic information; 3D view; checkCIF report

## Figures and Tables

**Table 1:** Hydrogen-bond geometry (Å, °) *Cg*3 is the centroid of C1–C6 ring

*D*—H⋯*A*	*D*—H	H⋯*A*	*D*⋯*A*	*D*—H⋯*A*
C5—H5⋯N1	0.93	2.47	2.827 (5)	103
C3—H3⋯*Cg*3^i^	0.93	2.91	3.5670 (1)	129

Symmetry code: (i) 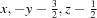.

**Table 2:** HUMO–LUMO energies and values of quantum chemical parameters (eV)

Property	Symbol and formula	Value
HOMO energy	E_H_ (eV)	−6.234
LUMO energy	E_L_ (eV)	−1.891
HOMO-1 energy	E_H-1_ (eV)	−6.552
LUMO+1 energy	E_L+1_ (eV)	−1.156
Energy gap 1	E_g1_ = (*E*_H_ - *E*_L_) (eV)	4.343
Energy gap 2	E_g2_ = (*E*_H-1_ - *E*_L+1_) (eV)	5.397
Global hardness	η = (*E*_L_ - *E*_H_)/2	2.172
Softness	ζ = 1/ 2η	0.230
Chemical potential	μ = (*E*_L_ + *E*_H_)/2	4.062
Electrophilicity	ψ = μ^2^/2η	3.799
Electronegativity	χ = -μ	−4.062

**Table 3:** Comparison of structural details in related imidazo[1,2-*a*]pyridinyl derivatives containing phenyl rings Dihedral angle 1 is the angle between the mean planes of imidazo[1,2-*a*]pyridinyl and phenyl rings. Two sets of dihedral angles 1 are stated for compounds HURZOL, MONREO, OMIDEV, RUJNEQ, TUZYEU, ZUSSAJ and VEGKAU because there are two mol­ecules in their asymmetric units.

Compound	*R*_1_	*R*_2_	Dihedral angle 1
**3-(Substituted)imidazo[1,2-*a*]pyridin­yl**			
AHOMIV (Liu *et al.*, 2015 ▸)	6-iodo-3-(methyl­sulfan­yl)-imidazo[1,2-*a*]pyridin­yl	phen­yl	27.0
BEGTUE (Nair *et al.*, 2012 ▸)	ethyl (imidazo[1,2-*a*]pyridin-3-yl)acetate	phen­yl	38.6
DABTEI (Koudad *et al.*, 2015 ▸)	6-nitro­imidazo[1,2-*a*]pyridinyl-3-carbaldehyde	4-meth­oxy­phen­yl	34.0
DIDZUO (Dey *et al.*, 2018 ▸)	3-chloro-7-methyl-imidazo[1,2*-a*]pyridin­yl	phen­yl	28.0
ECEGEA (Ma *et al.*, 2011 ▸)	ethyl 8-methyl-imidazo[1,2-*a*]pyridinyl-3-carboxyl­ate	phen­yl	44.2
HUPWIZ01 (Vega *et al.*, 2011 ▸)	*N*,*N*-dimethyl-2-(6-methyl-imidazo[1,2-*a*]pyridin-3-yl)acetamide	4-methyl­phen­yl	24.6
HURZOL (Yang *et al.*, 2015 ▸)	6-methyl­imidazo[1,2-*a*]pyridin-3-yl thio­cyanate	3-chloro­phen­yl	33.8, 27.7
KABMIM (Yang *et al.*, 2016 ▸)	6-methyl-3-nitro­soimidazo[1,2-*a*]pyridin­yl	3-chloro­phen­yl	6.8
MIXZOJ (Anaflous *et al.*, 2008*a* ▸)	*N*-(imidazo[1,2-*a*]pyridin-3-yl)acetamide	phen­yl	9.0
MIXZUP (Anaflous *et al.*, 2008*b* ▸)	imidazo[1,2-*a*]pyridinyl-3-carbaldehyde	phen­yl	28.6
MONREO (Velázquez-Ponce *et al.*, 2013 ▸)	3-nitro­soimidazo[1,2-*a*]pyridin­yl	phen­yl	17.4, 4.9
NOGRIM (Marandi *et al.*, 2014 ▸)	3-(*t*-butyl­amino)-imidazo[1,2-*a*]pyridinyl-8-carb­oxy­lic acid	3-nitro­phen­yl	16.8
OMIDEV (Samanta *et al.*, 2016 ▸)	3-iodo-8-methyl-imidazo[1,2-*a*]pyridin­yl	phen­yl	36.1, 34.4
QUQSEC (Ravi *et al.*, 2016 ▸)	6-methyl-3-(methyl­sulfan­yl)imidazo[1,2-*a*]pyridin­yl	4-chloro­phen­yl	38.1
RELQUW (Yan *et al.*, 2012 ▸)	8-methyl-3-nitro­imidazo[1,2-*a*]pyridin­yl	phen­yl	47.5
RUJNEQ (Li *et al.*, 2009 ▸)	imidazo[1,2-*a*]pyridinyl-3-carbaldehyde	4-chloro­phen­yl	34.6, 33.5
TUZYEU (Zhang *et al.*, 2016 ▸)	6-fluoro-3-nitro-imidazo[1,2-*a*]pyridin­yl	phen­yl	43.8, 37.9
UTITEX (Chunavala *et al.*, 2011 ▸)	ethyl 7-methyl­imidazo[1,2-*a*]pyridinyl-3-carboxyl­ate	phen­yl	39.6
YEDHIY (Georges *et al.*, 1993 ▸)	6-chloro-*N*,*N*-di­propyl­imidazo[1,2-*a*] pyridinyl-3-acetamide	4-chloro­phen­yl	15.2
ZUSSAJ (Xiao *et al.*, 2015 ▸)	3-chloro-imidazo[1,2-*a*]pyridin­yl	4-methyl­phen­yl	12.0, 0.3
**Non-3-(substituted)imidazo[1,2-*a*]pyridin­yl**			
BISDUF (Kutniewska *et al.*, 2018 ▸)	imidazo[1,2-*a*]pyridin­yl	2-hy­droxy-5-meth­oxy­phen­yl	6.0
BISFAN (Kutniewska *et al.*, 2018 ▸)	imidazo[1,2-*a*]pyridin­yl	2-hy­droxy-4-bromo­phen­yl	4.2
CAJTIQ (Aslanov *et al.*, 1983 ▸)	6-nitro-imidazo[1,2-*a*]pyridin­yl	phen­yl	3.3
FEMQOF (Kurteva *et al.* 2012 ▸)	imidazo[1,2-*a*]pyridin­yl	4-meth­oxy­phen­yl	12.5
JEBZEY (Zhu *et al.*, 2017 ▸)	imidazo[1,2-*a*]pyridin­yl	isophthalo­nitrile	46.4
MIQSUD (Jin *et al.*, 2019 ▸)	2-(imidazo[1,2-*a*]pyridin-5-yl)propan-2-ol	phen­yl	2.7
NAGGEH (Tafeenko *et al.*, 1996 ▸)	imidazo[1,2-*a*]pyridin­yl	phen­yl	4.4
NONFOM (Mutai *et al.*, 2008 ▸)	imidazo[1,2-*a*]pyridin­yl	2-hy­droxy­phen­yl	6.7
NUBVUD (Seferoğlu *et al.*, 2015 ▸)	7-methyl­imidazo[1,2-*a*]pyridin­yl	4-meth­oxy­phen­yl	0.7
NUBWAK (Seferoğlu *et al.*, 2015 ▸)	7-methyl-imidazo[1,2-*a*]pyridin­yl	phen­yl	5.3
QODZUG (Mutai *et al.*, 2014 ▸)	imidazo[1,2-*a*]pyridinyl-6-carbo­nitrile	2-hy­droxy­phen­yl	2.8
TIDVIN (Donohoe *et al.*, 2012 ▸)	6-bromo-imidazo[1,2-*a*]pyridin­yl	phen­yl	2.4
VEGKAU (Duan *et al.*, 2006 ▸)	imidazo[1,2-*a*]pyridin­yl	3-bromo-4-meth­oxy­phen­yl	12.2, 2.7
WUHKER (Aydıner *et al.*, 2015 ▸)	7-methyl­imidazo[1,2-*a*]pyridin­yl	4-chloro­phen­yl	9.1
ZUNVOV (Stasyuk *et al.*, 2016 ▸)	imidazo[1,2-*a*]pyridin­yl	2-hy­droxy-4-florophen­yl	3.2
ZUPCOE (Stasyuk *et al.*, 2016 ▸)	imidazo[1,2-*a*]pyridin­yl	2-hy­droxy-4-meth­oxy­phen­yl	5.8

**Table 4:** Experimental details

Crystal data	
Chemical formula	C_13_H_8_Br_2_N_2_
*M*_r_	352.03
Crystal system, space group	Orthorhombic, *P* *b* *c* *a*
Temperature (K)	293
*a*, *b*, *c* (Å)	14.1711 (4), 6.0546 (2), 27.7102 (8)
*V* (Å^3^)	2377.54 (12)
*Z*	8
Radiation type	Mo *K*α
μ (mm^−1^)	6.80
Crystal size (mm)	0.15 × 0.14 × 0.14

Data collection	
Diffractometer	Bruker Kappa APEXII CCD
No. of measured, independent and observed [*I* > 2σ(*I*)] reflections	41143, 3485, 1726
*R*_int_	0.100
(sin θ/λ)_max_ (Å^−1^)	0.704

Refinement	
*R*[*F* ^2^ > 2σ(*F* ^2^)], *wR*(*F* ^2^), *S*	0.047, 0.118, 1.00
No. of reflections	3485
No. of parameters	154
H-atom treatment	H-atom parameters constrained
Δρ_max_, Δρ_min_ (e Å^−3^)	0.88, −0.49

Computer programs: *APEX2* and *SAINT* (Bruker, 2016 ▸), *SHELXT* (Sheldrick, 2015*a* ▸), *SHELXL* (Sheldrick, 2015*b* ▸), *Mercury* (Macrae *et al.*, 2008 ▸), *PLATON* (Spek, 2009 ▸) and *publCIF* (Westrip, 2010 ▸).

## supplementary crystallographic information

### Crystal data

**Table:**

C_13_H_8_Br_2_N_2_	*D*_x_ = 1.967 Mg m^−^^3^
*M**_r_* = 352.03	Mo *K*α radiation, λ = 0.71073 Å
Orthorhombic, *P**b**c**a*	Cell parameters from 3485 reflections
*a* = 14.1711 (4) Å	θ = 2.1–30.1°
*b* = 6.0546 (2) Å	µ = 6.80 mm^−^^1^
*c* = 27.7102 (8) Å	*T* = 293 K
*V* = 2377.54 (12) Å^3^	Block, colourless
*Z* = 8	0.15 × 0.14 × 0.14 mm
*F*(000) = 1360	

### Data collection

**Table:**

Bruker Kappa APEXII CCD diffractometer	*R*_int_ = 0.100
ω and φ scan	θ_max_ = 30.1°, θ_min_ = 2.1°
41143 measured reflections	*h* = −19→19
3485 independent reflections	*k* = −8→8
1726 reflections with *I* > 2σ(*I*)	*l* = −39→38

### Refinement

**Table:**

Refinement on *F*^2^	0 restraints
Least-squares matrix: full	Hydrogen site location: inferred from neighbouring sites
*R*[*F*^2^ > 2σ(*F*^2^)] = 0.047	H-atom parameters constrained
*wR*(*F*^2^) = 0.118	*w* = 1/[σ^2^(*F*_o_^2^) + (0.0442*P*)^2^ + 2.6166*P*] where *P* = (*F*_o_^2^ + 2*F*_c_^2^)/3
*S* = 1.00	(Δ/σ)_max_ = 0.002
3485 reflections	Δρ_max_ = 0.88 e Å^−^^3^
154 parameters	Δρ_min_ = −0.49 e Å^−^^3^

### Special details

**Table:**

Geometry. All esds (except the esd in the dihedral angle between two l.s. planes) are estimated using the full covariance matrix. The cell esds are taken into account individually in the estimation of esds in distances, angles and torsion angles; correlations between esds in cell parameters are only used when they are defined by crystal symmetry. An approximate (isotropic) treatment of cell esds is used for estimating esds involving l.s. planes.

### Fractional atomic coordinates and isotropic or equivalent isotropic displacement parameters (Å^2^)

**Table:**

	*x*	*y*	*z*	*U*_iso_*/*U*_eq_	
Br2	0.15325 (4)	0.05914 (9)	0.27691 (2)	0.0700 (2)	
Br1	0.12673 (4)	0.88905 (10)	0.68410 (2)	0.0740 (2)	
N2	0.1377 (2)	0.3517 (6)	0.40975 (12)	0.0449 (8)	
N1	0.0885 (2)	0.6735 (6)	0.44091 (12)	0.0484 (9)	
C7	0.1238 (3)	0.5400 (6)	0.47646 (14)	0.0401 (9)	
C1	0.1234 (3)	0.7762 (8)	0.62067 (15)	0.0482 (11)	
C11	0.1297 (3)	0.2554 (7)	0.32808 (15)	0.0498 (11)	
C4	0.1241 (3)	0.6173 (7)	0.52687 (15)	0.0424 (9)	
C12	0.0872 (3)	0.4597 (8)	0.31737 (16)	0.0571 (12)	
H12	0.070603	0.493692	0.285760	0.069*	
C8	0.1549 (3)	0.3438 (8)	0.45819 (15)	0.0479 (10)	
H8	0.182324	0.228190	0.475266	0.058*	
C3	0.1597 (3)	0.4933 (7)	0.56424 (16)	0.0495 (10)	
H3	0.185335	0.355055	0.557750	0.059*	
C6	0.0866 (3)	0.9064 (7)	0.58414 (16)	0.0539 (11)	
H6	0.061410	1.044944	0.590752	0.065*	
C13	0.0707 (3)	0.6058 (8)	0.35300 (15)	0.0561 (12)	
H13	0.041977	0.740195	0.346048	0.067*	
C2	0.1582 (3)	0.5687 (8)	0.61068 (16)	0.0532 (11)	
H2	0.180652	0.480001	0.635558	0.064*	
C5	0.0885 (3)	0.8244 (7)	0.53745 (15)	0.0479 (10)	
H5	0.065063	0.911265	0.512485	0.057*	
C9	0.0968 (3)	0.5559 (7)	0.40055 (15)	0.0442 (10)	
C10	0.1555 (3)	0.2005 (7)	0.37352 (15)	0.0492 (11)	
H10	0.184202	0.065712	0.380142	0.059*	

### Atomic displacement parameters (Å^2^)

**Table:**

	*U*^11^	*U*^22^	*U*^33^	*U*^12^	*U*^13^	*U*^23^
Br2	0.0853 (4)	0.0701 (4)	0.0545 (3)	0.0001 (3)	0.0150 (3)	−0.0104 (2)
Br1	0.0737 (4)	0.1022 (4)	0.0460 (3)	−0.0056 (3)	0.0062 (2)	−0.0110 (3)
N2	0.039 (2)	0.047 (2)	0.049 (2)	−0.0024 (16)	0.0010 (15)	0.0053 (16)
N1	0.051 (2)	0.050 (2)	0.044 (2)	0.0047 (17)	0.0001 (16)	0.0062 (17)
C7	0.032 (2)	0.042 (2)	0.045 (2)	0.0013 (19)	−0.0038 (17)	0.0074 (18)
C1	0.041 (2)	0.063 (3)	0.040 (2)	−0.008 (2)	0.0008 (18)	0.004 (2)
C11	0.051 (3)	0.052 (3)	0.046 (2)	−0.002 (2)	0.007 (2)	−0.002 (2)
C4	0.030 (2)	0.047 (2)	0.051 (2)	−0.0038 (18)	−0.0002 (18)	0.005 (2)
C12	0.070 (3)	0.060 (3)	0.041 (2)	0.002 (2)	0.007 (2)	0.013 (2)
C8	0.043 (2)	0.055 (3)	0.046 (2)	0.002 (2)	−0.0061 (19)	0.006 (2)
C3	0.046 (2)	0.045 (2)	0.058 (3)	0.008 (2)	−0.001 (2)	0.008 (2)
C6	0.051 (3)	0.049 (3)	0.062 (3)	0.003 (2)	0.004 (2)	−0.001 (2)
C13	0.067 (3)	0.057 (3)	0.044 (2)	0.009 (2)	0.005 (2)	0.008 (2)
C2	0.047 (3)	0.066 (3)	0.046 (2)	0.007 (2)	−0.0042 (19)	0.008 (2)
C5	0.044 (2)	0.051 (3)	0.048 (2)	0.006 (2)	−0.0076 (19)	0.013 (2)
C9	0.044 (2)	0.041 (2)	0.048 (2)	0.0035 (19)	0.0013 (18)	0.0050 (19)
C10	0.047 (3)	0.042 (2)	0.059 (3)	0.002 (2)	0.002 (2)	0.000 (2)

### Geometric parameters (Å, º)

**Table:**

Br2—C11	1.880 (4)	C4—C5	1.383 (6)
Br1—C1	1.886 (4)	C12—C13	1.346 (6)
N2—C8	1.365 (5)	C12—H12	0.9300
N2—C10	1.382 (5)	C8—H8	0.9300
N2—C9	1.389 (5)	C3—C2	1.366 (6)
N1—C9	1.331 (5)	C3—H3	0.9300
N1—C7	1.369 (5)	C6—C5	1.386 (6)
C7—C8	1.364 (6)	C6—H6	0.9300
C7—C4	1.473 (6)	C13—C9	1.401 (6)
C1—C2	1.378 (6)	C13—H13	0.9300
C1—C6	1.385 (6)	C2—H2	0.9300
C11—C10	1.353 (6)	C5—H5	0.9300
C11—C12	1.407 (6)	C10—H10	0.9300
C4—C3	1.375 (6)		
			
C8—N2—C10	131.2 (4)	C2—C3—C4	121.4 (4)
C8—N2—C9	106.6 (3)	C2—C3—H3	119.3
C10—N2—C9	122.2 (4)	C4—C3—H3	119.3
C9—N1—C7	104.8 (3)	C1—C6—C5	118.1 (4)
C8—C7—N1	111.4 (4)	C1—C6—H6	120.9
C8—C7—C4	128.9 (4)	C5—C6—H6	120.9
N1—C7—C4	119.7 (4)	C12—C13—C9	120.1 (4)
C2—C1—C6	120.5 (4)	C12—C13—H13	119.9
C2—C1—Br1	120.6 (3)	C9—C13—H13	119.9
C6—C1—Br1	119.0 (4)	C3—C2—C1	120.0 (4)
C10—C11—C12	121.9 (4)	C3—C2—H2	120.0
C10—C11—Br2	119.9 (3)	C1—C2—H2	120.0
C12—C11—Br2	118.2 (3)	C4—C5—C6	122.0 (4)
C3—C4—C5	118.0 (4)	C4—C5—H5	119.0
C3—C4—C7	122.8 (4)	C6—C5—H5	119.0
C5—C4—C7	119.2 (4)	N1—C9—N2	111.0 (3)
C13—C12—C11	119.8 (4)	N1—C9—C13	130.6 (4)
C13—C12—H12	120.1	N2—C9—C13	118.3 (4)
C11—C12—H12	120.1	C11—C10—N2	117.6 (4)
C7—C8—N2	106.1 (4)	C11—C10—H10	121.2
C7—C8—H8	127.0	N2—C10—H10	121.2
N2—C8—H8	127.0		
			
C9—N1—C7—C8	0.9 (5)	C6—C1—C2—C3	2.4 (7)
C9—N1—C7—C4	−178.9 (4)	Br1—C1—C2—C3	−176.7 (3)
C8—C7—C4—C3	1.1 (6)	C3—C4—C5—C6	−1.0 (6)
N1—C7—C4—C3	−179.1 (4)	C7—C4—C5—C6	179.9 (4)
C8—C7—C4—C5	−179.8 (4)	C1—C6—C5—C4	1.2 (6)
N1—C7—C4—C5	0.0 (6)	C7—N1—C9—N2	−0.7 (4)
C10—C11—C12—C13	−0.5 (7)	C7—N1—C9—C13	178.7 (5)
Br2—C11—C12—C13	−178.9 (4)	C8—N2—C9—N1	0.3 (5)
N1—C7—C8—N2	−0.8 (5)	C10—N2—C9—N1	−178.8 (4)
C4—C7—C8—N2	179.0 (4)	C8—N2—C9—C13	−179.2 (4)
C10—N2—C8—C7	179.3 (4)	C10—N2—C9—C13	1.7 (6)
C9—N2—C8—C7	0.3 (4)	C12—C13—C9—N1	179.3 (5)
C5—C4—C3—C2	1.5 (6)	C12—C13—C9—N2	−1.4 (7)
C7—C4—C3—C2	−179.4 (4)	C12—C11—C10—N2	0.8 (6)
C2—C1—C6—C5	−1.8 (6)	Br2—C11—C10—N2	179.2 (3)
Br1—C1—C6—C5	177.3 (3)	C8—N2—C10—C11	179.8 (4)
C11—C12—C13—C9	0.8 (7)	C9—N2—C10—C11	−1.4 (6)
C4—C3—C2—C1	−2.2 (7)		

### Hydrogen-bond geometry (Å, º)

*Cg*3 is the centroid of C1–C6 ring

**Table:**

*D*—H···*A*	*D*—H	H···*A*	*D*···*A*	*D*—H···*A*
C5—H5···N1	0.93	2.47	2.827 (5)	103
C3—H3···*Cg*3^i^	0.93	2.91	3.5670 (1)	129

Symmetry code: (i) *x*, −*y*−3/2, *z*−1/2.

### Summary of short interatomic contacts (Å)

**Table:**

Contact	Distance	symmetry
H5···H5	2.24	*1-x,2-y,-z*
Br1···H12	3.01	*x,3/2-y,-1/2+z*
